# Non-extraction Orthodontic Treatment Protocol of Moderate Crowding

**DOI:** 10.7759/cureus.37483

**Published:** 2023-04-12

**Authors:** Sumukh Nerurkar, Ranjit Kamble, Japneet Kaiser, Jeni Mathew

**Affiliations:** 1 Department of Orthodontics, Datta Meghe Institute of Higher Education and Research, Sharad Pawar Dental College, Wardha, IND

**Keywords:** iotn, ipion, preventive orthodontics, interceptive orthodontics, extraction of teeth, crowding

## Abstract

Dental crowding is referred to as the swarming of teeth, mainly due to the discrepancy between the size of the jaw bases and that of the teeth. When the amount of space required for the teeth is more than that in the jaws, it leads to crowding. The prevalence of crowding has now increased to almost 30-60%. It can be classified into mild, moderate, and severe according to the amount of overlap. Depending on the severity of the crowding, the decision of extraction is made. The given case presents a non-extraction protocol for treating moderate crowding. The present case report explains the non-extraction treatment of moderate crowding using inter-proximal stripping.

## Introduction

Dental crowding is a discrepancy between jaw and teeth size, which leads to misalignment [1]. It is seen when the size of the teeth is more compared to that of the jaws. According to a study conducted by Ahamed et al. in Tamil Nadu, the increase in consumption of processed food has increased the prevalence of dental crowding, which has increased to almost 60% [2]. The most common etiology of crowding is proximal caries in the primary dentition, which leads to mesial shifting of the posterior teeth, causing a decrease in jaw size. Other causes of crowding include genetics, deleterious habits, poor dental hygiene, and cleft lip and palate [3]. Crowding is classified into three groups, such as mild, moderate, and severe, according to the amount of overlap present [4].

Treatment includes creating space using extraction, interproximal stripping, expansion, distalization of molars, derotation, and uprighting teeth [5-8]. This decision about creating space is made according to the severity of crowding. Severe crowding calls for the extraction of one or more teeth; moderate crowding is treated using interproximal stripping or extraction of a single incisor, whereas mild crowding is treated using stripping [9]. The given case explains the method of treating moderate crowding without undergoing extraction.

## Case presentation

**Figure 1:**
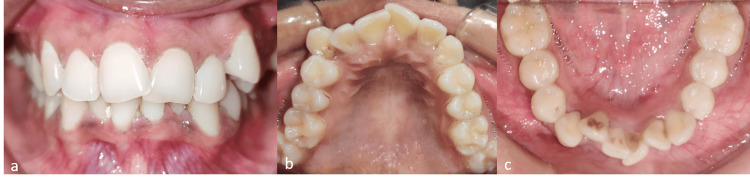
Intraoral pre-treatment photographs. (a) Frontal view showing maxillary and mandibular crowding. (b) Maxillary occlusal view showing upper crowding. (c) Mandibular occlusal view showing lower crowding.

A 20-year-old female came to the clinic with a complaint of malaligned teeth. The extraoral features include a straight profile, average nasolabial angle, competent lips, bilaterally symmetrical face, mesocephalic head form, mesoprosopic facial form, straight facial divergence, and an average clinical Frankfort-mandibular plane angle [10]. The intraoral examination showed a normal alveolar ridge, average shape and size of the tongue, normal height of the labial and buccal vestibule, and normal health of the gingiva. The hard tissue examination showed that all the permanent teeth were present, with an overjet of 2mm and an overbite of 1.5mm, and the maxillary anterior teeth were upright concerning the basal bone. The molar and canine relationship was class I on either side - 4mm crowding was seen in the upper arch and 6mm crowding was seen in the lower arch. The lower right lateral incisor was rotated. An orthopantomogram (OPG) ruled out the presence of any supernumerary teeth. The treatment objective was to align the upper and lower teeth, to maintain the molar and canine relationship, allowing for proclination of maxillary anterior teeth and maintaining the normal overjet and overbite. Figure 1 depicts the pre-treatment intraoral photographs of the patient.

**Figure 2:**
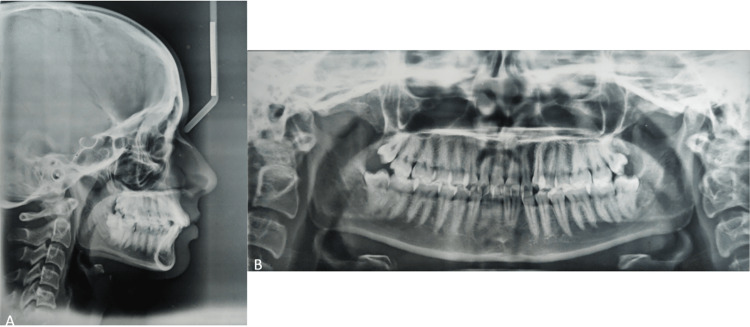
(A) Lateral cephalogram and (B) orthopantomogram depicting the absence of supernumerary or impacted teeth.

The cephalometric analysis showed that the inclination of the maxillary and mandibular anteriors was normal (upper 1 to NA: 22° and lower 1 to NB: 25°). The nasolabial angle was 91°. Ashley Howe’s analysis concluded that it is a borderline case; thus, the interproximal reduction was considered [11]. The anterior Bolton ratio was 79.6%, and the overall ratio was 92.1%. Both ratios infer that there is tooth material excess in the mandibular anterior segment [12]. Figure 2 depicts the pre-treatment lateral cephalogram and OPG.

Treatment plan 

The treatment plan included bonding of the maxillary and mandibular arches followed by initial leveling and alignment. The proclination of the anteriors would be prevented by a passive lace back from the molar to the canine, and the creation of space will be done by interproximal stripping. A total of 0.25mm reduction was done for each contact surface in the anterior region from 13 to 23 and 33 to 43, and a 0.4mm reduction was done in 14, 24, 34, and 44 regions, which accounts for 3mm of the total reduction in the mandibular tooth material. The stripping was done using diamond discs.

**Figure 3:**
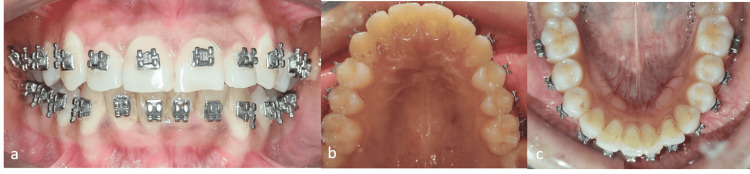
Intraoral posttreatment photograph. (a) Frontal view showing corrected crowding. (b) Maxillary occlusal view showing aligned arch. (c) Mandibular occlusal view showing aligned arch.

The treatment was commenced after the maxillary and mandibular arches were bonded using 0.022″ MBT brackets. The initial leveling and alignment of the maxillary arch were done using a 0.0155″ coaxial wire, which was followed by 0.016″ × 0.022″ and 0.017″ × 0.025″ nickel-titanium (NiTi) wires [13]. The mandibular teeth were aligned using the same wire sequence, which was done after the interproximal stripping from 33 to 43. The desensitizing paste was advised to the patient to prevent any sensitivity following the interproximal stripping [6]. The mandibular anterior teeth were prevented from proclining with the help of passive lace back from the molars to the canines. The rotated 42 was derotated using a rotational wedge on a 0.012″ NiTi wire piggybacked onto a 0.019″ × 0.025″ stainless steel wire [14]. Currently, the arches are well aligned, and the mild discrepancies in the mandibular anterior teeth are being treated. The post-alignment radiograph showed that there was no significant increase in the maxillary and mandibular incisor proclination (upper 1 to NA: 26° and lower 1 to NB: 27°). The total treatment duration was nine months. Cases with rotated teeth are complicated to retain due to the rebound effect of the gingival fibers on the tooth. Thus, such cases must be given permanent retention or under circumferential supracrestal fiberotomy (CSF). To prevent relapse, a permanent retainer is planned immediately after debonding [15]. Figure 3 depicts the post-treatment intraoral photographs. 

## Discussion

Dental crowding has become a very commonly seen dental malocclusion owing to the reduced fibrous food in the diet. Earlier, this dental crowding was prevented by attrition of the teeth that occurred due to the raw fibrous diet of humans. Begg explained this theory in his article “stone age man’s dentition” [16]. Highly processed food has also increased the prevalence of caries in the primary dentition, which causes a decrease in arch length. Although not the most common reason for seeking orthodontic treatment, crowding accounts for a bulk of patients that want to undergo treatment. Appropriate preventive and interceptive procedures can also be advocated to reduce the chances of the development of malocclusion [17]. Correct diagnosis and treatment planning are required to treat the malocclusion and, more importantly, retain it. The correction rotations must be retained using permanent retention or must undergo CSF to reduce the effect of gingival fibers. Nerurkar and Kamble confirmed the role of over-retained teeth and the presence of supernumerary teeth in causing crowding [18]. Lucas in his study confirmed that the main cause of dental crowding in modern humans is the consumption of highly processed food [19]. Yitschaky et al. confirmed with their study that for every 1mm alleviation of crowding, there is a proclination of 0.5° and 0.2mm [20]. The major advantage of using this technique is the avoidance of extraction, but in some cases, it may cause sensitivity.

## Conclusions

The present case explained the treatment of moderate crowding without undergoing extraction. The paradigm has now shifted from extraction to non-extraction protocol. In cases where the discrepancy is not severe enough to undergo extraction, interproximal stripping is an excellent alternative to correct the discrepancy. The present case explains that stripping can be done in some cases without causing significant proclination and, at the same time, improving the overall aesthetics of the patient.
